# Dysautonomia Differentially Influences the Effect of Affective Pain Perception on Quality of Life in Parkinson's Disease Patients

**DOI:** 10.1155/2016/3067426

**Published:** 2016-04-28

**Authors:** D. Rada, J. Seco, E. Echevarría, B. Tijero, L. C. Abecia, J. C. Gómez-Esteban

**Affiliations:** ^1^Department of Preventive Medicine and Public Health, University of the Basque Country, Paseo de la Universidad No. 7, 1006 Vitoria-Gasteiz, Spain; ^2^Institute of Biomedicine (IBIOMED), University of León, Campus Vegazana, s/n, 24071 León, Spain; ^3^University of the Basque Country, Vitoria-Gasteiz, Spain; ^4^Department of Physiology, University of the Basque Country, CIBERSAM, Paseo de la Universidad No. 7, 1006 Vitoria-Gasteiz, Spain; ^5^Neurodegenerative Unit, Biocruces Research Institute, Cruces Plaza, 48903 Bilbao, Spain

## Abstract

*Background*. Our aim was to evaluate the real effect of dysautonomic symptoms on the influence of affective pain perception on quality of life in PD patients.* Methods*. An observational cross-sectional study was carried out using 105 Parkinson's disease (PD) patients of the Movement Disorders Unit, Hospital de Cruces (Bilbao, Spain) [men 59 (56.2%), women 46 (43.85%)]. Statistical analysis was made in order to evaluate the possible association of pain with life quality.* Results*. Quality of life measured by PDQ-39 (Parkinson's Disease Questionnaire for quality of life) was statistically associated with affective dimension of pain (PRIA, affective pain rating index). However, the influence of this dimension on PDQ-39 was different in the specific case of PD patients that experimented a high score (>12) in SCOPA-AUT (Scale for Outcomes in PD-Autonomic scale).* Conclusions*. These results confirm the effect of affective perception of pain in life quality of PD patients, indicating the critical role of autonomic symptoms in the modulation of the influence of pain on quality of life and showing the possible utility of dysautonomia as clinical prognostic indicator of quality of life in PD patients affected by pain.

## 1. Introduction 

Parkinson's disease (PD) is a chronic neurodegenerative illness caused by degeneration of the dopaminergic nigrostriatal pathway of the central nervous system, which implies impairment in quality of life (QoL). Motor [1] and nonmotor symptoms (NMS), such as cognitive, mood and autonomic disorders [2, 3], are present in PD patients. Neuropsychiatric disorders, sleep disorders, pain, motor complications, and depression are the variables with the greatest effect on QoL in PD patients [4]. In fact, some of these symptoms, such as constipation, hyposmia and REM sleep behavior disorders (RBD), have been proposed as possible early biomarkers [5].

Dopamine signaling alterations can modulate the experience of pain [6]. PD can cause motor and autonomic alterations, as well as pain [7, 8]. However, although several studies have focused on this topic [9], it is frequently undertreated [10]. Several types of pain can coexist in these patients [11], conditioning their clinical management [12]. Thus, although musculoskeletal, radicular-neuropathic, and dystonic pain, as well as akathisia, can be observed, central primary pain is considered a specific symptom of this disease [13]. It has been demonstrated that pain in PD patients is associated with female gender [14], as well as with the presence of depression [15], but not with age or disease duration, leading to impairment in QoL. However, not only pain but also dysautonomic symptoms have been recently shown to be associated with the prevalence of depression and anxiety in older PD patients, which are frequently untreated [16].

Sensory and autonomic dysfunction are an important prodromal feature of PD and can result in a major disability and seriously impact the health-related QoL in these patients [17]. However, although it has been demonstrated that nonmotor symptoms can induce a severe impairment in health-related QoL [18], treatments are usually focused on motor symptoms. In fact, their frequency, treatment rates, and impact on HRQoL in the early motor phase are unclear. Thus, both pain and depression have been linked to dysfunction of serotonin and noradrenaline in this disease [19]. The increased prevalence of depression in PD patients has been associated with diminished locus coeruleus projections to limbic structures in studies that measured catecholaminergic transporter binding (11[C]RTI-32 PET). Reduced noradrenergic innervation impairs reflex cardiovascular responses and is the main cause of autonomic failure in many PD patients. Potent inhibitors of serotonin and noradrenaline, like duloxetine, have shown efficacy on pain in Parkinson's disease, confirming the role of both neurotransmitters in the genesis of these symptoms [20, 21]. Dysautonomia is a frequent problem in PD patients [22], even before the development of motor symptoms, leading to cardiovascular, gastrointestinal, urological, sexual, and thermoregulatory alterations, with a considerable impairment in QoL [23].

Taken into account the association between the nociceptive and autonomic nervous systems [24], our aim was to evaluate the real effect of dysautonomic symptoms on the influence of affective pain perception on QoL in PD patients.

## 2. Methods 

### 2.1. Patients

A total of 105 patients [men 59 (56.2%), women 46 (43.85%)] who fulfilled the UK Parkinson's disease Society Brain Bank criteria for the clinical diagnosis of PD (aged between 43 and 87 years) were included in the study (see Appendix A). Pharmacological treatment of all patients was in accordance with the ordinary protocols applied in their reference hospital (see Appendix B). All of them were patients of the Movement Disorders Unit, Hospital de Cruces (Basque Health Service/Osakidetza, Bilbao, Spain), admitted during the period September 2011–April 2013 (mean age at initial diagnosis: 59.98 ± 9.17 years). This is a university hospital that receives referrals of all individuals with PD from a geographic area of about 500,000 inhabitants, in the north of Spain.

### 2.2. Procedure

After clinical examination, staging of the disease was based upon the findings of the clinical examination and in accordance with the present standards with respect to motor symptoms [Modified Hoehn and Yahr Staging [25] and Unified Parkinson's Disease Rating Scale UPDRS [26]] (see Appendix C).

Scores on clinical scales for evaluation of motor and autonomic symptomatology, as well as several aspects of pain perception and QoL, were the main outcome measure of the study. Thus, the battery of clinical scales used in this study included the Parkinson's Disease Questionnaire for quality of life (PDQ-39), adapted to Spanish language and culture (PDQ-39 Spanish version, PDQ-39SV) in order to evaluate HRQoL in PD patients [27].

Pain perception was evaluated using the McGill Pain Questionnaire (McGill Pain Index) (MPQ) in the categories sensory (MPQ-S), affective (MPQ-A), miscellaneous (MPQ-M), pain rating index (PRI), and present pain intensity (PPI). The affective dimension score of the McGill Pain Questionnaire can serve as a useful index of the overall affective status of pain patients and given this interpretation the dimension has good construct validity [28].

Autonomic symptoms were evaluated using the Scale for Outcomes in PD-Autonomic scale (SCOPA-AUT) classified in six dimensions. This scale has good content validity and has good known-groups validity and discriminates between control, mild, moderate, and severe PD groups [29].

### 2.3. Statistical Analysis

All statistical analyses were performed using the IBM SPSS® Statistics for Windows (version 22) (SPSS, Chicago, IL). The Shapiro-Wilk test was used to check whether the quantitative variables were normally distributed. The baseline characteristics of the sample were summarized using descriptive statistics (means and standard deviation for quantitative variables, and percentages in the case of qualitative ones) (see Appendix A).

With respect to the bivariant analysis, the breakpoint for comparisons in the variable quality of life (PDQ-39SV) was taken at the value of 30. Thus, the sample was divided into a group of patients with good life quality (PDQ-39SV < 30) and another group with impaired life quality (PDQ-39SV > 30). In this case, data of 8 patients were missing due to the fact that questionnaires were not correctly fulfilled. Differences between these two groups were analyzed for the case of several variables, such as pain perception, motor and autonomic symptomatology, sex, age, medication, and time of evolution. The breakpoint for comparisons in the variable pain (PRI) was taken at the categories 0 (including the items no pain + mild) and 1 (including the items discomforting + distressing + horrible + excruciating). Thus, the sample was divided into a group of patients with acceptable pain perception and other group integrated by patients that suffered considerable or even strong pain intensity. Differences between these two groups were analyzed for the case of several variables, such as life quality, motor and autonomic symptomatology, sex, age, medication, and time of evolution. The breakpoint for comparisons in the case of the variable SCOPA-AUT was taken at the value of 12. Thus, the sample was divided in a group of patients with high intensity of dysautonomic symptoms (SCOPA-AUT > 12) and other group with low intensity (SCOPA-AUT < 12). Differences between these two groups were analyzed for the case of several variables, such as pain perception, motor and autonomic symptomatology, sex, age, medication, and time of evolution. Breakpoints for PDQ-39 and SCOPA-AUT were based on the median values of sample distributions. In the bivariant analysis, Student's *t*-test was used for mean comparison. For nonparametric distributions, Mann-Whitney test was used. In the case of qualitative variables, Pearson's chi square test was used.

Correlation analysis among different aspects of MPQ and PDQ-39 was carried out in patients with high and low SCOPA-AUT scores. Pearson's coefficient or Spearman's coefficient for nonparametric distributions were used.

Linear regression models were built to evaluate the influence of pain (MPQ) (including subparts such as affective, sensory, and miscellaneous) and other independent variables (*X*) such as gender, age, medication, time of evolution, and autonomic and motor symptomatology on quality of life (PDQ-39) (dependent variable, *Y*). With respect to predictive models, *R* ^2^ coefficient was obtained. Durbin-Watson test was calculated in order to discard a hypothetical nonparametric distribution of residuals. In addition, the presence of collinearity was discarded using variance inflation factor and condition index. All *p* values presented were two tailed and *p* < 0.05 was considered statistically significant.

### 2.4. Ethical Approach

The research was conducted in accordance with the 2000 version of the Declaration of Helsinki. The study was approved by the Ethics and Clinical Research Boards of the Hospital de Cruces (Spain) and Institute Biocruces. The reference of the Ethical Commission decision was March 31st of 2009. Patients, parents, or legal representatives gave written informed consent and all participants assented to participate in the study.

## 3. Results 

Clinically considerable differences within the group of patients with good quality of life (PDQ-39SV < 30), with respect to those with impaired life quality (PDQ-39SV > 30), were observed in several variables, particularly in SCOPA-AUT and PRIT (Table 1). Also, differences were observed when comparing the group of patients with acceptable pain perception to those who suffered considerable or even strong pain intensity, especially in the variables SCOPA-AUT (*p* < 0.01) and PDQ-39SV (*p* = 0.033) (Table 2). Moreover, the group of patients with low score in SCOPA-AUT (<12) was compared to the group of patients with a high score in SCOPA-AUT (>12), showing differences in several variables analyzed, such as PDQ-39SV (*p* < 0.01) and PRIT (*p* < 0.01) (Table 2).

A significant positive correlation between PRIT and PRIA as well as between PRIA and PDQ-39 in the selected PD patients was found. However, the magnitude of these correlations changed considerably when they were calculated in patients with a score in SCOPA-AUT lesser than or equal to 12, with respect to the case of the group of patients with a score in SCOPA-AUT higher than 12. Thus, in the case of the group of PD patients with a low SCOPA-AUT score, correlation between PRIT and PRIA was of 84% (*p* < 0.01), whereas in the group of PD patients with a high score in SCOPA-AUT it was of 43% (*p* = 0.003). Also, correlation of PRIA with PDQ-39 was of 13% in the group of low score SCOPA-AUT patients (*p* = 0.347), whereas in the case of high score SCOPA-AUT patients it was of 27% (*p* = 0.075) (Table 3).

Linear regression confirmed that PRIA was statistically associated with PDQ-39SV (Table 4). However, the influence of PRIA on PDQ-39SV was considerably different when the linear regression model was built using exclusively those PD patients that experimented a high score in SCOPA-AUT scale (>12), with respect to the case of the model elaborated specifically in the group of patients with a low score in SCOPA-AUT (≤12) (Table 4).

## 4. Discussion 

The reported results confirmed a relation between the affective perception of pain (PRIA) and quality of life (PDQ-39SV) in PD patients. Moreover, we could observe that this influence of affective perception of pain on QoL was considerably higher in the case of those PD patients that suffered a high degree of autonomic symptoms, indicating that they are a determinant factor that modifies the impact of pain in QoL in PD patients. Therefore, our results indicate that dysautonomia could have a role as clinical prognostic indicator of QoL in PD patients affected by pain and should be taken into account in their clinical management.

A significant positive correlation between PRIT and PRIA, as well as between PRIA and PDQ-39SV, was observed in the selected patients in the present study. However, these correlations changed considerably when they were calculated in patients with a high degree of dysautonomic symptomatology, with respect to the case of the group of patients with a lesser degree of dysautonomia, indicating the contribution of dysautonomia in pain perception and central nervous system processing of pain in PD patients [30].

As it could be expected, bivariant analysis showed some differences within the group of patients with good QoL, with respect to those with impaired quality, in the degree of dysautonomia, all components of pain perception and motor symptomatology, and daily equivalent levodopa dosage, as well as in disease duration, indicating not only the natural clinical evolution of PD but also the important influence of dysautonomia on the QoL of patients [31].

Differences in several clinical variables were also observed when comparing the group of patients with acceptable pain perception with respect to those who suffered considerable or even strong pain intensity. Thus, the differences in dysautonomia and QoL reported here could indicate not only the well-known effect of pain on QoL [11, 12, 18] but also the coexistence of dysautonomia and pain impairment in PD patients [16].

Patients with low levels of autonomic symptoms were compared with those suffering a high degree of dysautonomia. In this case, statistical significant differences were found in several variables, such as QoL and all components of pain perception and motor symptomatology, as well as in daily equivalent levodopa dosage, indicating that dysautonomic PD patients not only have, as could be expected, a lesser degree of QoL [11, 12, 16, 18] but also display more motor symptoms and a higher perception of pain.

## 5. Conclusions

Although the results reported here confirm the effect of affective perception of pain in life quality of PD patients, this influence was considerably higher when it was evaluated only in a selected group of PD patients that suffered from a high degree of dysautonomia, indicating the critical role of autonomic symptoms in the modulation of the influence of pain on QoL and showing the possible utility of dysautonomia as clinical prognostic indicator of QoL in PD patients affected by pain.

## Figures and Tables

**Table 1:** Bivariant analysis by quality of life (PDQ-39SV > 30 versus PDQ-39SV ≤ 30) *n* = 97.

	PDQ-39SV ≤ 30 (*n* = 49)	PDQ-39SV > 30 (*n* = 48)	*p*
	Mean (SD)	Mean (SD)	
Age	67.57 (8.94)	70.46 (8.51)	0.270
Sex (women%)	36.7	52.1	0.128
Age at PD diagnosis	59.54 (9.65)	60.13 (8.84)	0.914
Disease duration (years)	8.03 (5.04)	10.33 (5.48)	0.014
SCOPA-AUT	9.49 (5.03)	17.96 (7.51)	<0.001
UPDRS I	1.82 (2.09)	4.04 (2.35)	<0.001
UPDRS II	10.19 (4.84)	15.60 (5.39)	<0.001
UPDRS III	27.35 (9.01)	35.42 (9.69)	<0.001
UPDRS IV	2.10 (2.05)	4.89 (3.60)	<0.001
PRIS	6.16 (5.10)	9.6 (3.89)	0.001
PRIM	1.47 (1.80)	2.38 (1.88)	0.010
PRIA	1.16 (1.51)	2.42 (1.87)	<0.001
PRIT	8.80 (7.52)	14.40 (5.74)	<0.001
Daily equivalent levodopa dosage (mg)	388.02 (305.90)	666.88 (298.78)	<0.001

**(a):** 

	Without pain (*n* = 20)	With pain (*n* = 85)	*p*
	Mean (SD)	Mean (SD)	
Age	66.10 (8.93)	69.73 (8.48)	0.091
Sex (women%)	15	50.6	0.004
Age at PD diagnosis	58.01 (8.78)	60.45 (9.25)	0.289
Disease duration (years)	8.08 (5.26)	9.29 (5.51)	0.363
SCOPA-AUT	7.45 (4.03)	15.06 (7.39)	<0.001
UPDRS I	2.80 (1.99)	2.92 (2.56)	0.872
UPDRS II	10.00 (4.14)	13.48 (5.77)	0.005
UPDRS III	28.20 (7.54)	32.20 (10.32)	0.106
UPDRS IV	1.75 (1.55)	3.85 (3.27)	0.005
PDQ-39	20.28 (13.34)	32.81 (21.85)	0.033
Daily equivalent levodopa dosage (mg)	419.34 (317.43)	540.08 (329.07)	0.149

**(b):** 

	Low SCOPA (≤12) *n* = 47	High SCOPA (>12) *n* = 58	*p*
	Mean (SD)	Mean (SD)	
Age	67.95 (9.37)	70.39 (7.54)	0.413
Sex (women%)	41.4	46.8	0.577
Age at PD diagnosis	59.2 (9.82)	61 (8.31)	0.332
Disease duration (years)	8.76 (5.47)	9.4 (5.48)	0.476
PDQ-39	22.30 (16.38)	40.77 (21.92)	<0.001
UPDRS I	3.34 (2.47)	2.53 (2.39)	0.068
UPDRS II	15.15 (5.21)	10.95 (10.00)	<0.001
UPDRS III	35.94 (8.93)	27.79 (9.27)	<0.001
UPDRS IV	4.26 (3.51)	2.79 (2.63)	0.018
PRIS	6.10 (4.95)	9.91 (4.04)	<0.001
PRIM	1.34 (1.68)	2.60 (1.94)	0.001
PRIA	1.12 (1.35)	2.53 (1.93)	<0.001
PRIT	8.57 (6.92)	15.04 (5.86)	<0.001
Daily equivalent levodopa dosage (mg)	586.97 (48.75)	461.18 (315.94)	0.087

**Table 3:** Correlation coefficients among PRI (total), PRI (sensory, miscellaneous, or affective), and PDQ-39SV.

	Low SCOPA (≤12)		High SCOPA (>12)	
	*R*	*p*	*R*	*p*
PRIT versus PRIS	0.945	<0.001	0.856	<0.001
PRIT versus PRIM	0.695	<0.001	0.595	<0.001
PRIT versus PRIA	0.848	<0.001	0.431	0.003
PRIT versus PDQ-39	0.129	0.353	0.118	0.452
PRIS versus PDQ-39	0.077	0.582	0.041	0.796
PRIM versus PDQ-39	0.18	0.193	0.039	0.802
PRIA versus PDQ-39	0.131	0.347	0.274	0.075

**(a):** 

Variable	*B*	SE	*p*	95% CI	
(Durbin-Watson 1.57, *R* ^2^ 0.26).					
PRIA	3.973	1.068	<0.001	1.853	6.093

**(b):** 

Variable	*B*	SE	*p*	95% CI	
*SCOPA ≤ 12* (Durbin-Watson 1.62, *R* ^2^ 0.21).					
PRIA	0.059	1.635	0.972	−3.226	3.343
Equivalent medication (mg)	0.024	0.007	0.001	0.01	0.038

*SCOPA > 12 *(Durbin-Watson, 1.33, *R* ^2^ 0.22)					
PRIA	4.135	1.576	0.012	0.949	7.321
Equivalent medication (mg)	0.018	0.009	0.065	−0.001	0.037

**Table 5:** Baseline characteristics (*n* = 105).

	Mean (SD)
Age	69.04 (8.64)
Age at PD diagnosis	59.98 (9.17)
Disease duration (years)	9.06 (5.46)
Pain intensity (visual scale 0–10)	4.88 (3.11)
Motor situation (UPDRS III)	31.44 (9.95)

	Percentage (%)

Sex (women%)	43.8
Incapacitating dyskinesia (%)	14.4
Painful dyskinesia (%)	6.7
Morning dystonia (%)	19.2
Anorexia, nausea, or vomiting (%)	16.3
Sleeping disorders (%)	53.8

**Table 6:** UPDRS subscales (*n* = 105).

	Mean (SD)	Median
UPDRS I	2.90 (2.45)	2
UPDRS II	12.81 (5.64)	12
UPDRS III	31.44 (9.95)	30
UPDRS IV	3.44 (3.12)	3

**Table 7:** Medication (mg).

	*n*	Mean (SD)	Median
Total equivalent medication	93	579.30 (291.83)	540
Equivalent levodopa	93	541.94 (265.16)	500
Standard levodopa	91	504.40 (238.36)	500
Controlled-release levodopa	19	315.79 (173.25)	200
Ropinirole	36	13.47 (5.93)	13.5
Pramipexole	27	1.93 (0.68)	2
Amantadine	8	212.50 (35.36)	200
Selegiline	2	7.5 (3.54)	7.5
Apomorphine	3	63.33 (52.17)	76
Entacapone	32	725 (207.91)	800
Rotigotine	14	8.14 (4.26)	8
Trihexifenidilo	1	4	4

## Appendix

### A. Baseline Characteristics

See Table 5.

### B. UPDRS Subscales

See Table 6.

### C. Medication (mg)

See Table 7.

## Abbreviations

PD: Parkinson's disease
MPQ: McGill Pain Questionnaire
PRIS: Sensitive pain rating index
PRIA: Affective pain rating index
PRIE: Evaluative pain rating index
PRIM: Miscellaneous pain rating
PRIT: Total pain rating index
SCOPA-AUT: Scale for Outcomes in PD-Autonomic
UPDRS: Unified Parkinson's Disease Rating Scale
MMSE: Mini Mental State Examination
PDQ-39: Parkinson's Disease Questionnaire for quality of life
PDQ-39SV: Parkinson's Disease Questionnaire for quality of life Spanish version
QoL: Quality of life.
